# In your eyes only: deficits in executive functioning after frontal TMS reflect in eye movements

**DOI:** 10.3389/fnbeh.2014.00007

**Published:** 2014-01-27

**Authors:** Mathias Lüthi, Katharina Henke, Klemens Gutbrod, Thomas Nyffeler, Silvia Chaves, René M. Müri

**Affiliations:** ^1^Perception and Eye Movement Laboratory, Department of Neurology and Clinical Research, University Hospital Bern InselspitalBern, Switzerland; ^2^Center for Cognition, Learning, and Memory, University of BernBern, Switzerland; ^3^Division of Experimental Psychology and Neuropsychology, Department of Psychology, University of BernBern, Switzerland; ^4^Center of Neurology and Neurorehabilitation, Luzerner KantonsspitalLuzern, Switzerland

**Keywords:** eye movements, executive functions, theta burst TMS, dorsolateral prefrontal cortex, medial frontal cortex

## Abstract

This study investigated the roles of the right and left dorsolateral prefrontal (rDLPFC, lDLPFC) and the medial frontal cortex (MFC) in executive functioning using a theta burst transcranial magnetic stimulation (TMS) approach. Healthy subjects solved two visual search tasks: a number search task with low cognitive demands, and a number and letter search task with high cognitive demands. To observe how subjects solved the tasks, we assessed their behavior with and without TMS using eye movements when subjects were confronted with specific executive demands. To observe executive functions, we were particularly interested in TMS-induced changes in visual exploration strategies found to be associated with good or bad performance in a control condition without TMS stimulation. TMS left processing time unchanged in both tasks. Inhibition of the rDLPFC resulted in a decrease in anticipatory fixations in the number search task, i.e., a decrease in a good strategy in this low demand task. This was paired with a decrease in stimulus fixations. Together, these results point to a role of the rDLPFC in planning and response selection. Inhibition of the lDLPFC and the MFC resulted in an increase in anticipatory fixations in the number and letter search task, i.e., an increase in the application of a good strategy in this task. We interpret these results as a compensatory strategy to account for TMS-induced deficits in attentional switching when faced with high switching demands. After inhibition of the lDLPFC, an increase in regressive fixations was found in the number and letter search task. In the context of high working memory demands, this strategy appears to support TMS-induced working memory deficits. Combining an experimental TMS approach with the recording of eye movements proved sensitive to discrete decrements of executive functions and allows pinpointing the functional organization of the frontal lobes.

## Introduction

Cognitive processes are well known to influence eye movements (Kowler, 2011). Similarly, complex visual tasks require specialized visual exploration strategies (Hodgson et al., 2000). Detailed analysis of these strategies may provide more information on cognitive functioning than conventional measures (Hodgson et al., 2002). Eye movements, in comparison to manual behavior are more automatically performed and therefore a more direct and unbiased measure of ongoing mentation (Hannula et al., 2010). While individuals are able to shift their attention without moving their eyes, they cannot move their eyes while paying attention to another location (Wright and Ward, 2008). Hence, a gaze shift indexes an attentional shift (Lamers and Roelofs, 2011).

Because of these characteristics, eye movements can be particularly helpful in the observation of efficient and strategic exploration behavior, abilities commonly referred to as executive functions. These functions allow us to organize our thoughts and actions in a goal-directed way, create a plan, initiate and monitor its execution, while keeping track of the progress. They inhibit inappropriate thoughts and actions and adapt our behavior flexibly to a changing environment (e.g., Jurado and Rosselli, 2007). Indeed, eye movements have been previously shown to be suitable to observe planning, working memory, attention to task relevant and irrelevant information as well as shifting attention (e.g., Hodgson et al., 2000, 2002; Mennie et al., 2007; Godijn and Theeuwes, 2012; Wass et al., 2011).

When assessing executive functions, a fundamental problem is the low process-behavior correspondence: While an executive deficit may lead to a myriad of behavioral difficulties, a specific behavior can be generated by a variety of impaired executive (and non-executive) processes (Tranel et al., 1994; Burgess, 1997). This problem can in part be attributed to the use of global performance measures such as time or errors. These measures do not directly reveal the cognitive deficit causing the observed impairment and are therefore insufficient starting points for the investigation of brain–behavior relationships (Stuss et al., 2002, 2005; Wölwer and Gaebel, 2002).

This study set out to investigate and compare the functional roles of the right and left dorsolateral prefrontal cortex (rDLPFC, lDLPFC) and the medial frontal cortex (MFC) in executive functions. Even though executive functions are known to have their neural basis in the frontal cortex (Alvarez and Emory, 2006), a functional specification of the rDLPFC, lDLPFC and the MFC in efficient behavior using an experimental, comparative approach is still missing. To this end, we chose transcranial magnetic stimulation (TMS). When studying the brain–behavior relationship, TMS avoids some of the methodological shortcomings of descriptive lesion and correlative imaging studies (Rorden and Karnath, 2004). Compared to lesion studies, TMS can induce more homogenous changes in the same, predefined brain area in a larger group of healthy subjects. The theta burst stimulation (TBS) protocol used in this study is known to induce a long lasting functional inhibition of the stimulated cortical area (Nyffeler et al., 2006; Hubl et al., 2008).

We used two computerized visual search tasks based on parts A and B of the Trail Making Test (Reitan and Wolfson, 1985), one of the most frequently used measures of executive functioning (Rabin et al., 2005). For this task, the analysis of time measurements did not prove to be the most useful method of categorizing performance in frontal patients (Stuss et al., 2001). The first task in our study was a number search task, which required subjects to search for (but not connect) ascending numbers. In the second task, ascending number and letter sequences had to be processed (but not connected) in an alternating fashion (1-A-2-B…). The two tasks differ in their cognitive demands. The number search task requires going through a sequence of numbers, an easy and highly overlearned task. Because of its low executive demands, we considered this task a suitable paradigm for investigating basic executive functions such as paying attention to task relevant stimuli and mentally anticipating future stimuli. In contrast, the number and letter search task is more challenging. It requires mentally switching between numbers and letters as well as keeping track of the actual position within both sequences. Therefore, the number and letter search task was considered a suitable tool to observe TMS-induced deficits in attentional switching and working memory.

Here, we explore the functional roles of the rDLPFC, lDLPFC and the MFC in efficient and goal directed visual behavior. In our quest to observe executive functions “at work”, we were interested in how inhibitory TMS over the three frontal regions influence visual strategies associated with good (i.e., fast) performance as well as poor (i.e., slow) performance in control conditions of two tasks with low and high cognitive demands. As the rDLPFC, lDLPFC and the MFC have all been linked to executive functioning, we expected a functional inhibition of these areas to affect the use of efficient visual strategies. We expected TMS-induced changes in eye movement parameters previously described to reflect executive functioning. Specifically, we were interested in planning (i.e., anticipatory fixations, see Mennie et al., 2007), response selection (i.e., fixations on task relevant vs. irrelevant information, see Hodgson et al., 2000, 2002), attentional switching (i.e., fixations on relevant information during switch implementation, see Chevalier et al., 2010) and working memory (i.e., regressive fixations when faced with working memory demands, see Kemper et al., 2004). TMS-induced changes in good visual strategies could either reflect executive malfunctioning or attempts to compensate deficient executive functioning when confronted with specific cognitive task demands.

In this study, we used TMS-induced changes in efficient visual strategies during specific cognitive demands as a tool to pinpoint executive functions associated with the rDLPFC, lDLPFC and the MFC.

## Materials and methods

### Subjects

Each of the three TMS groups consisted of 18 naive, healthy subjects. Six males and 12 females, mean age 32.0 ± 11.2 (mean ± *SD*) years, were part of the rDLPFC stimulation group. Eight males and 10 females, aged 31.1 ± 6.7 years took part in the MFC stimulation group. The lDLPFC group consisted of 8 males and 10 females with a mean age of 31.7 ± 7.5 years. Subjects were recruited from employees of the University of Berne and the University Hospital Bern and paid a cinema voucher for participation. All subjects had normal or corrected-to-normal vision. All but three subjects were right-handed. All left-handed subjects showed evidence of left hemispheric language dominance in the “DLCV-108” dichotic listening test (Hugdahl and Andersson, 1986). The study was in accordance with the latest Declaration of Helsinki and approved by the ethics committee of the Canton of Berne, Switzerland. Subjects gave written informed consent before participation.

### Stimuli

**Figure 1:**
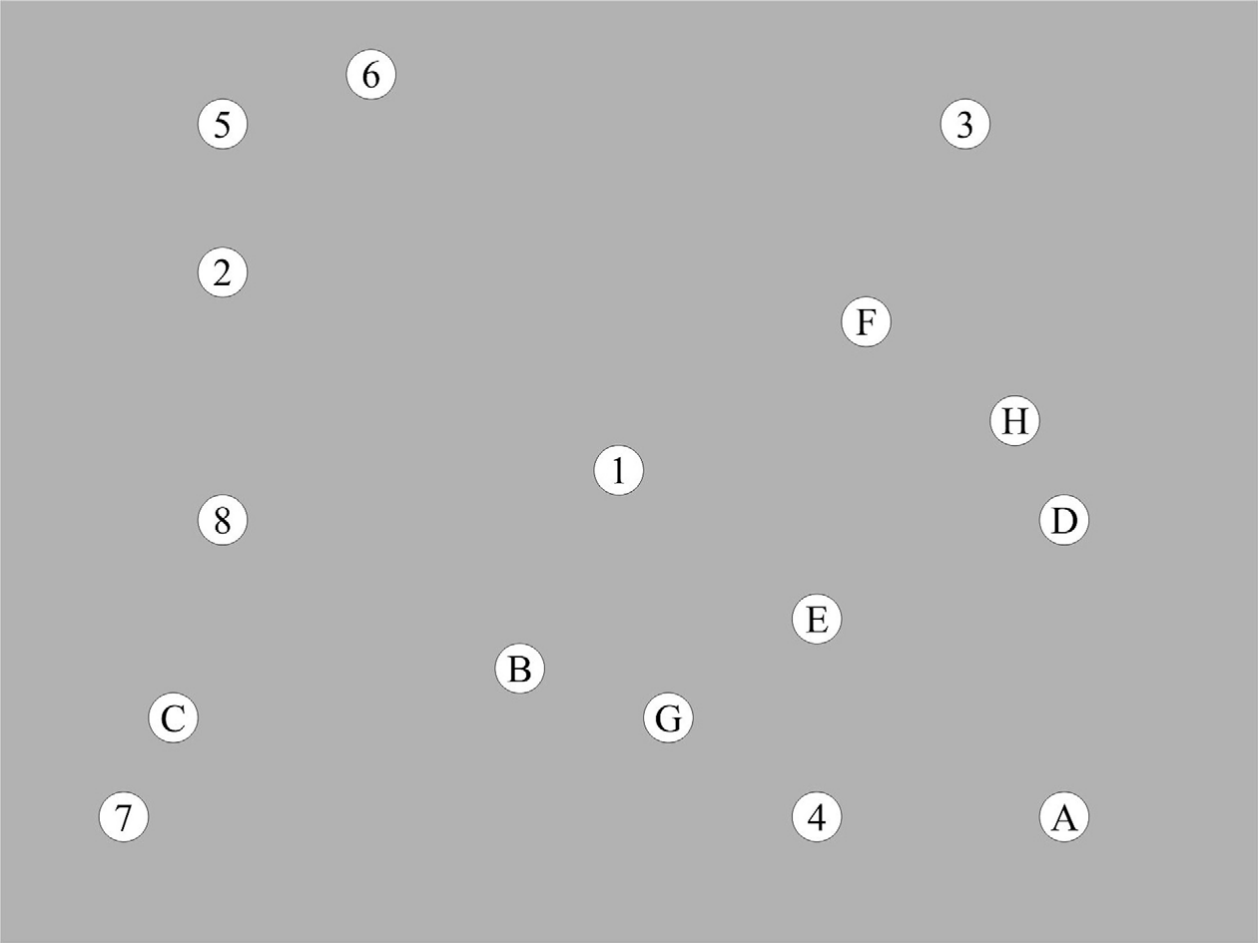
**Example of a number and letter search task stimulus display**.

In both tasks, subjects were instructed to search for and click on 16 stimuli on a computer screen in ascending order as fast as possible and without errors. This was done by moving a mouse cursor over a stimulus on the screen and pressing the mouse key. In the number search task, the stimuli were numbers 1–16. In the second task, the stimuli consisted of numbers and letters, which had to be processed in an alternating manner (1-A-2-B… until the letter H was reached). The numbers and letters appeared in white dots before a gray background (Figure 1).

To minimize learning of the spatial positions of stimuli over experimental trials, four different spatial layouts of stimuli were used in consecutive order. To ensure the same search difficulty, the spatial distance between two consecutive stimuli at all positions in the sequence was held constant across the four different stimulus layouts.

Before the experiment, subjects performed two practice trials to gain familiarity with the tasks. Experimental trials started with the appearance of the visual search display that was preceded by a fixation cross in the middle of the screen and at the same location as the first stimulus. If a mouse key press occurred after the cursor was placed over the correct stimulus, a clicking noise was played automatically and all stimuli in the white dots briefly disappeared, leaving the white dots empty for 250 ms. If a mouse click was made at the wrong location, e.g., if stimuli were not clicked in the correct order, subjects heard a buzzer sound which indicated that the error had to be corrected. Errors were thus accounted for by processing time. Clicking on stimuli left them unmarked, which required participants to mentally keep track of the progress. An experimental trial ended when all 16 stimuli were clicked in the correct order.

The stimuli were presented on a 20-inch TFT display (41 × 31 cm), with a 1600 × 1200 pixel resolution, 32 bit color depth and a refreshing rate of 60 Hz. Subjects were positioned at a distance of 71.5 cm from the screen, resulting in a visual angle of 32 (width) × 24 (height) degrees. Screen displays consisted of 16 stimuli that were 1.7 cm in diameter, corresponding to 1.3° visual angle.

### Design

In each of the three TMS groups, subjects completed two experimental sessions: one session with TMS, and one control session without stimulation (TMS is a within subjects factor). The order of the TMS and control session was randomized. Each session consisted of six number search task trials and six number and letter search trials that were performed in alternating order (12 trials per experimental session, and a total of 24 trials in the two sessions of the experiment). To minimize learning effects, the time interval between the two sessions was at least 1 week.

Processing time (cumulative mouse reaction time) per trial was used to assess global performance. To assess visual behavior, we measured the number of stimulus fixations, the number of fixations on the next stimulus in the sequence (anticipatory fixations, e.g., fixations on stimulus “3” while searching for “2” in the number search task, or fixations on stimulus “5” while searching for “E” in the number and letter search task) as well as the fixation number on the previous stimulus in the sequence (regressive fixations, e.g., fixations on stimulus “1” while searching for “2” in the number search task, or fixations on “A” while searching for “2” in the number and letter search task) in relation to the total number of fixations (i.e., % stimulus-, % anticipatory-, and % regressive fixations) in experimental trials.

The experiment was designed to ensure that a sufficient amount of fixations for valid statistical analysis could be measured over the course of the six experimental trials per condition and subject.

### TMS procedure

A TMS stimulator (MagPro, Medtronic Functional Diagnostics, Skovlunde, Denmark) was used to generate repetitive biphasic magnetic pulses. Before experimental sessions, a continuous train of theta burst TMS (TBS) was applied (600 pulses in total; a burst of three pulses with 30-Hz was repeated at intervals of 100 ms; Nyffeler et al., 2006) over the right or left DLPFC or the MFC. Because of the extended size and the lack of precisely defined target regions within the DLPFC (Petrides and Pandya, 1999), a round coil (Magnetic Coil MC-125, Medtronic) was used for DLPFC stimulation. The focality of the round coil (as opposed to a “figure 8” coil) is more in line with the size of the stimulated region. To stimulate the MFC, which includes the anterior cingulate cortex (ACC), a “figure 8” coil (Magnetic Coil MC-B70, Medtronic) was used to avoid stimulating more lateral parts of the frontal cortex. During stimulation, the handle of the coil pointed backwards (45° angle to the sagittal line).

The dorsolateral prefrontal cortex was located as previously described (Müri et al., 1996). In brief, stimulating the right or left motor cortex with single pulses determined the individual motor threshold by corresponding muscle twitching of the subject's relaxed small hand muscles. The coil was then moved 5 cm anteriorly. TBS was delivered at 90% of the individual subject's hand motor rest threshold. The mean stimulation intensity in the rDLPFC sample was 35.0 (*SD* 3.6), and 31.6 (*SD* 3.1) percent of the maximum output for the lDLPFC.

The MFC stimulation site was located as described by Hadland et al. (2001). To account for the greater distance of the MFC (including the ACC) from the surface of the brain, stimulation intensity was set according to the foot motor threshold. First, the foot representation of the motor cortex (located at approximately the same depth than the MFC) was located by placing the “figure 8” coil over the midline and searching for the point of maximum evoked movement in the outstretched feet with minimum stimulation strength. Then the stimulation intensity was gradually reduced to find the subject's active motor threshold. The site of stimulation was located over the midline four cm anterior to the subjects' foot area. TBS was delivered at 90% of the individual subjects' active foot motor threshold. The mean stimulation intensity was 46.3 (*SD* 6.0) percent of the maximum output.

### Data acquisition and eye movement recording

Data acquisition started typically 4 min after stimulation and was typically completed within 20 min after stimulation. Eye movements were assessed using a video based infrared eyetracking system (HiSpeed™, SensoMotoric Instruments GmbH, Teltow, Germany), at a sampling rate of 240 Hz and a spatial resolution of 0.5 – 1.0° (manufacturer's specification: <0.025°). To identify fixations, the minimum fixation duration was set at 80 ms and the dispersion threshold was 150 pixels. To avoid head movements subjects were made to position their chin on a rest. Before the experiment, a 13 point calibration session was performed. If necessary, this was repeated during the course of the experiment. To account for eccentric viewing and measuring inaccuracy, fixations within a 1.7 cm or 1.3° visual angle radius from the center of a stimulus (double the radius of the stimulus) were considered as fixations on this stimulus, avoiding overlapping stimulus fixation areas between stimuli. To allow for drift compensation, fixation data were manually recalibrated after the experiment for best possible stimulus fit. The stimuli presentation and recording of mouse clicks was performed with E-Prime software (Schneider et al., 2002).

## Results

We calculated repeated measures analysis of variances (ANOVAs) in all TMS groups (rDLPFC, MFC, lDLPFC). To detect within-subjects differences between the control and TMS condition (TMS is a within subjects factor), processing time and eye movement parameters (% stimulus fixations, % anticipatory fixations, % regressive fixations) served as dependent variables.

To identify good and bad visual strategies, we calculated Pearsons correlations between eye movement parameters (% stimulus fixations, % anticipatory fixations, % regressive fixations) and processing time in control condition trials. The level of significance was set at 0.05.

### TMS effects on general eye movement parameters

To investigate a possible effect of stimulation on eye movement regions, we tested for TMS-induced changes in general eye movement parameters: TMS over the rDLPFC, lDLPFC, and over the MFC did not alter the number of fixations [right DLPFC: *F*_(1, 17)_ = 0.39; left DLPFC: *F*_(1, 17)_ = 0.21; MFC: *F*_(1, 17)_ = 0.19] or the mean fixation duration [right DLPFC: *F*_(1, 17)_ = 0.05; left DLPFC: *F*_(1, 17)_ = 1.81; MFC: *F*_(1, 17)_ = 1.75]. This makes it very unlikely that the observed effects in our study are due to the stimulation of the frontal eye fields or the supplementary eye fields.

### Visual strategies associated with good and poor performance

Looking ahead was identified as a good visual strategy in both tasks. The percentage of anticipatory fixations was negatively correlated with processing time in control conditions of the number search task (*r* = −0.65, *p* < 0.01) and the number and letter search task (*r* = −0.44, *p* < 0.01). Conversely, looking back was a bad visual strategy in the number and letter search task, as the percentage of regressive fixations was positively correlated with processing time in control conditions (*r* = 0.47, *p* < 0.01). No bad visual strategy was identified in the number search task.

### TMS effects in the number search task

Table 1 provides an overview of descriptive statistics in the number search task.

**Table 1:** **Descriptive statistics (mean, *SE*) in the right dorsolateral prefrontal cortex group (*n* = 18), MFC group (*n* = 18) and left dorsolateral prefrontal cortex group (*n* = 18) for control and TMS conditions in number search task trials**.

**Number search task**	**Right DLPFC group (Control/TMS)**		**MFC group(Control/TMS)**		**Left DLPFC group (Control/TMS)**	
Processing time per trial (sec)	28.9±1.7	28.9±1.5	27.2±1.6	27.6±1.6	26.4±1.6	26.4±1.3
Number of fixations per trial	88.6±4.9	88.4±4.3	86.3±5.2	90.7±5.5	85.4±4.6	88.2±4.5
Fixation duration (msec)	307.6±9.4	304.4±9.7	306.5±9.6	298.3±8.3	290.8±10.6	295.0±8.5
% Stimulus fixations	**60.0 ± 1.1**	**54.9 ± 2.2**	61.3±0.9	60.2±1.3	56.1±2.5	55.6±2.4
% Regressive fixations	0.43±0.1	0.33±0.1	0.56±0.1	0.57±0.1	0.54±0.1	0.46±0.1
% Anticipatory fixations	**7.4 ± 0.9**	**5.9 ± 0.8**	9.1±1.0	10.0±1.2	8.2±0.8	9.2±1.2

*Significantly different visual strategies are in bold*.

#### TMS effects on performance

Irrespective of stimulation site, TMS had no effects on processing time in the number search task [right DLPFC: *F*_(1, 17)_ = 0.005; left DLPFC: *F*_(1, 17)_ = 0.000; MFC: *F*_(1, 17)_ = 0.18]. Hence, TMS-induced deficits in neural computation did not reflect in a classical measure of task performance, but in a more subtle performance measure, namely exploration behavior.

#### TMS effects on visual strategies associated with good performance

**Figure 2:**
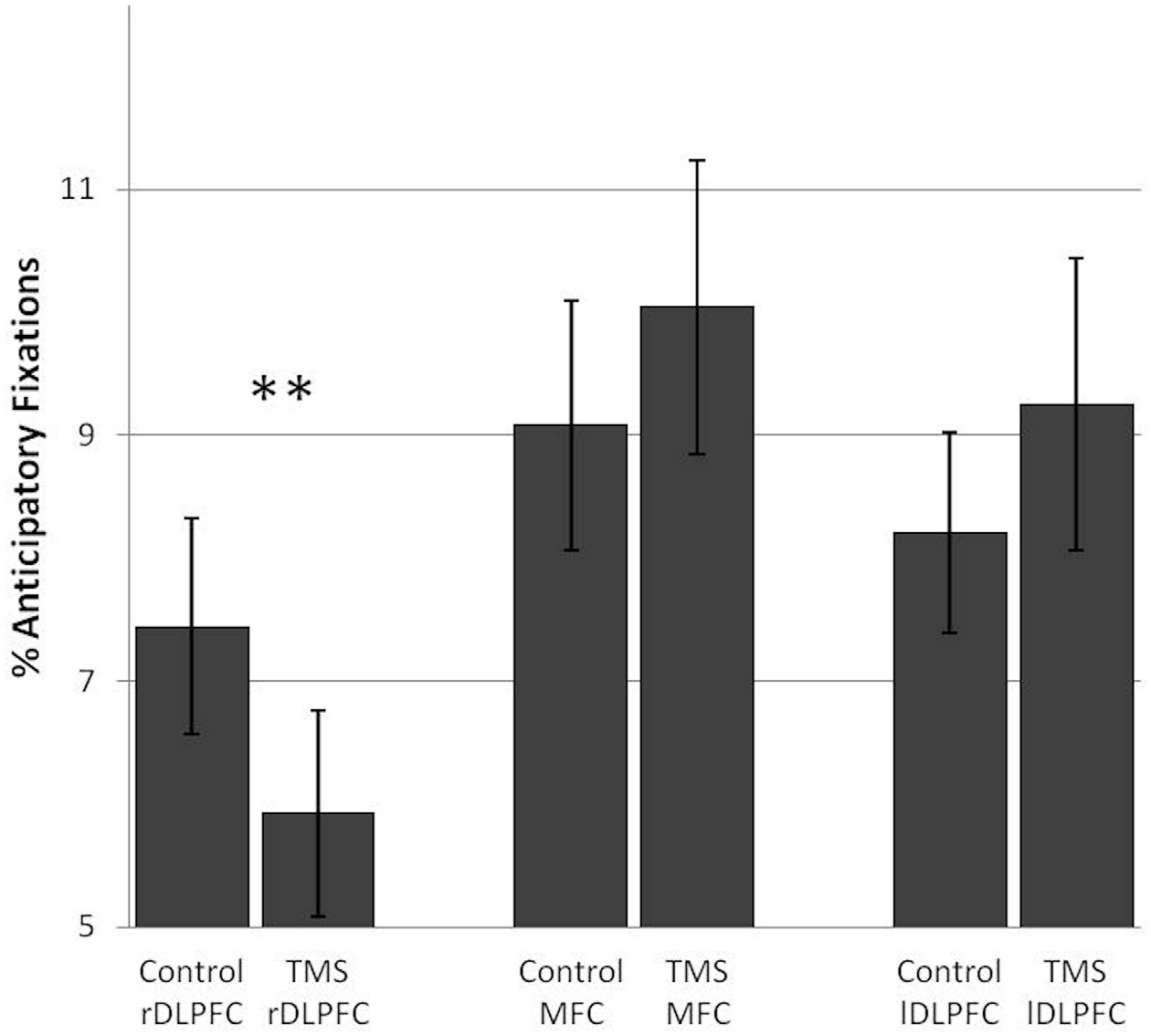
**TMS effects on the percentage of anticipatory fixations in the number search task**. A significant difference in anticipatory fixations between the control and TMS condition was found after stimulation over the right dorsolateral prefrontal cortex. Error bars indicate standard errors of the mean (SE). ^**^0.003.

TMS over the rDLPFC led to a highly significant decrease in anticipatory fixations [*F*_(1, 17)_ = 11.86, *p* = 0.003]. This effect was found in 83% of the subjects. No change in anticipatory fixations was found after TMS over the lDLPFC TMS [*F*_(1, 17)_ = 0.96] or the MFC [*F*_(1, 17)_ = 1.84] (Figure 2).

#### TMS effects on stimulus fixations

**Figure 3:**
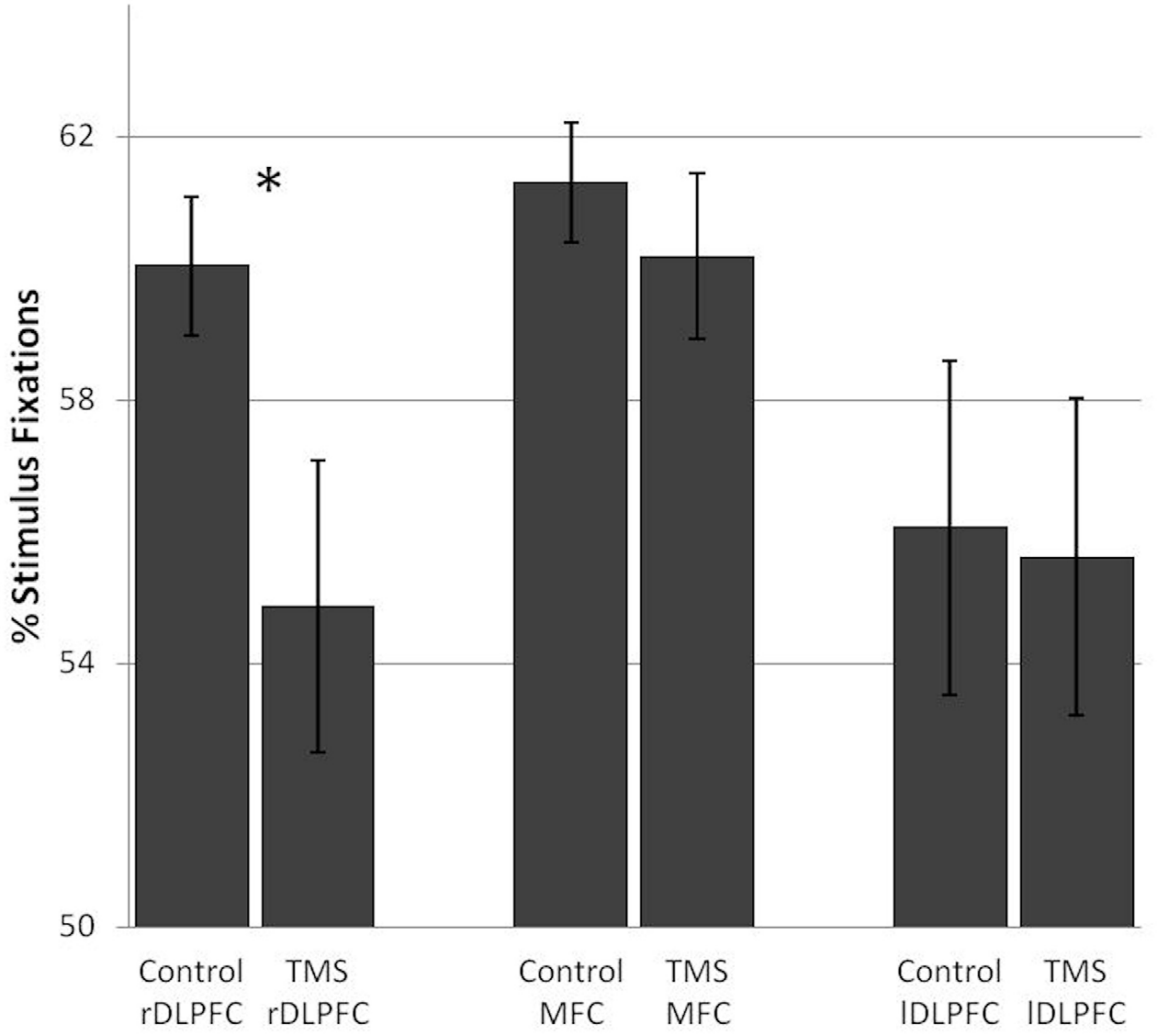
**TMS effects on the percentage of stimulus fixations in the number search task**. A significant difference in stimulus fixations between the control and TMS condition was found after stimulation over the right dorsolateral prefrontal cortex. Error bars indicate standard errors (SE). ^*^0.016.

After rDLPFC stimulation, a decrease in the number of fixations on stimuli and a concurrent increase in background fixations [*F*_(1, 17)_ = 7.19, *p* = 0.016] was found in over 70% of the subjects. No such pattern was found after lDLPFC [*F*_(1, 17)_ = 0.07] or MFC stimulation [*F*_(1, 17)_ = 0.64] (Figure 3).

In sum, functional inhibition of the rDLPFC led to a decrease in anticipatory fixations, a good visual strategy in a task with low executive demands, without affecting performance itself. This was paired with a general decrease in fixations on task relevant stimuli and a concurrent increase in background fixations, where only the moving mouse cursor was visible.

### TMS effects in the number and letter search task

Table 2 provides an overview of descriptive statistics in the number and letter search task.

**Table 2:** **Descriptive statistics (mean, *SE*) in the right dorsolateral prefrontal cortex group (*n* = 18), MFC group (*n* = 18) and left dorsolateral prefrontal cortex group (*n* = 18) for control and TMS conditions in number and letter search task trials**.

**Number and letter search task**	**Right DLPFC group (Control/TMS)**		**MFC group (Control/TMS)**		**Left DLPFC group (Control/TMS)**	
Processing time per trial (sec)	34.8±2.4	36.2±2.8	34.2±2.3	32.1±1.6	34.0±2.0	33.0±1.8
Number of fixations per trial	107.3±6.6	112.7±9.0	106.6±6.4	104.4±5.3	108.0±5.2	107.7±5.6
Fixation duration (msec)	291.0±8.1	292.6±9.4	291.5±7.6	295.8±7.6	276.2±8.6	286.3±6.8
% Stimulus fixations	57.0±1.4	55.4±2.0	59.4±1.1	61.0±1.2	54.5±2.8	56.7±2.1
% Regressive fixations	0.99±0.2	0.98±0.2	0.82±0.2	1.12±0.1	**0.83 ± 0.2**	**1.31 ± 0.2**
% Anticipatory fixations	3.9±0.5	3.8±0.6	**4.9 ± 0.7**	**6.5 ± 0.7**	**4.0 ± 0.4**	**5.4 ± 0.5**

*Significantly different visual strategies are in bold*.

#### TMS effects on performance

TMS over the rDLPFC or lDLPFC, or over the MFC had no effects on processing time in the number and letter search task [right DLPFC: *F*_(1, 17)_ = 0.85; left DLPFC: *F*_(1, 17)_ = 0.84; MFC: *F*_(1, 17)_ = 3.37].

#### TMS effects on visual strategies associated with good performance

**Figure 4:**
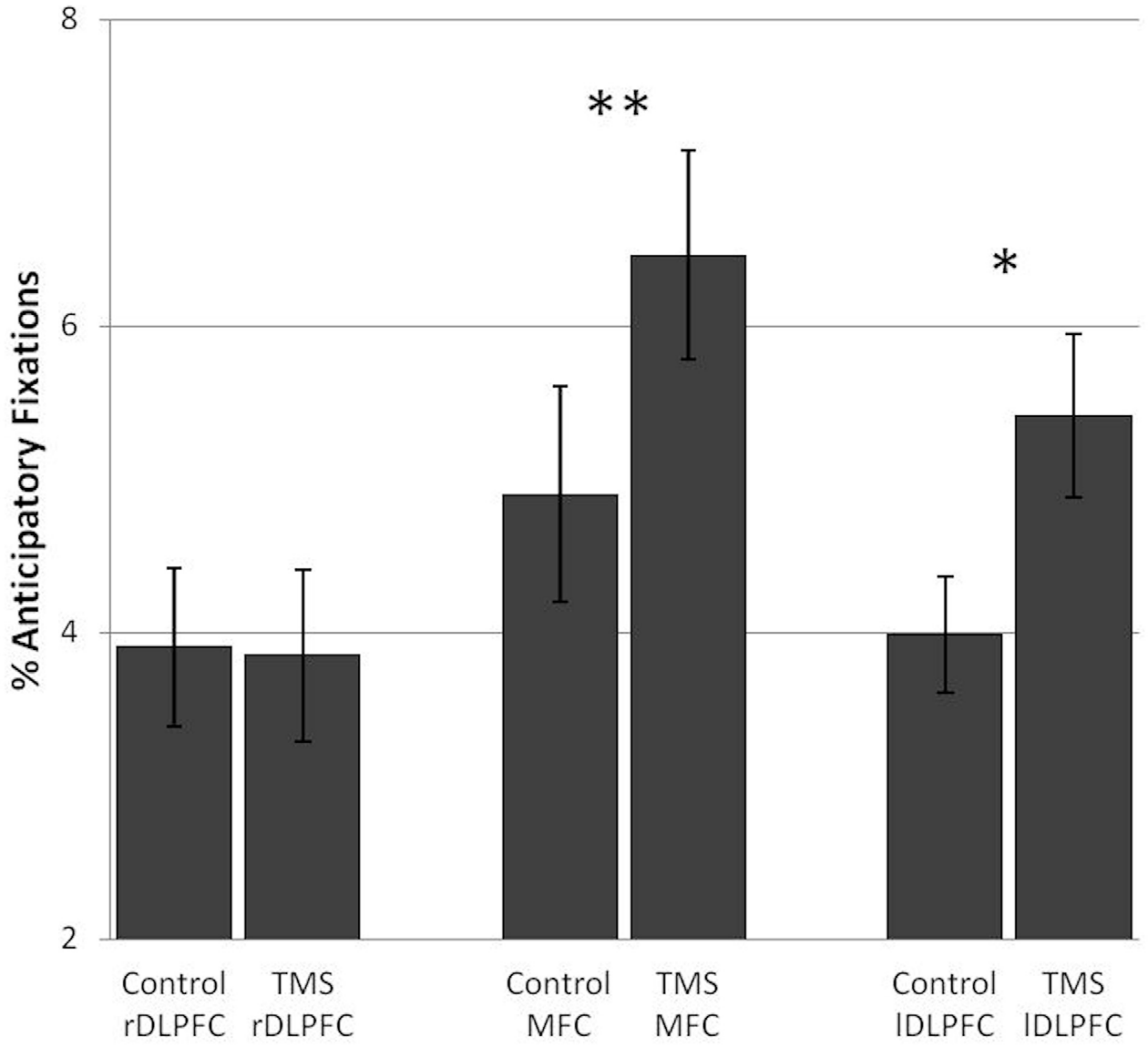
**TMS effects on the percentage of anticipatory fixations in the number and letter search task**. Significant differences in anticipatory fixations between the control and TMS conditions were found in the medial frontal cortex TMS group and the left dorsolateral prefrontal cortex TMS group. Error bars indicate standard errors of the mean (SE). ^*^0.015; ^**^0.007.

Subjects showed an increase in anticipatory fixations after TMS over the MFC [*F*_(1, 17)_ = 9.45, *p* = 0.007] as well as after TMS over the lDLPFC [*F*_(1, 17)_ = 7.27, *p* = 0.015]. In both TMS groups this was observed in the vast majority of subjects (72 and 78%, respectively). No TMS effects on anticipatory fixations were found in the rDLPFC TMS group [*F*_(1, 17)_ = 0.03]. (Figure 4).

#### TMS effects on visual strategies associated with poor performance

**Figure 5:**
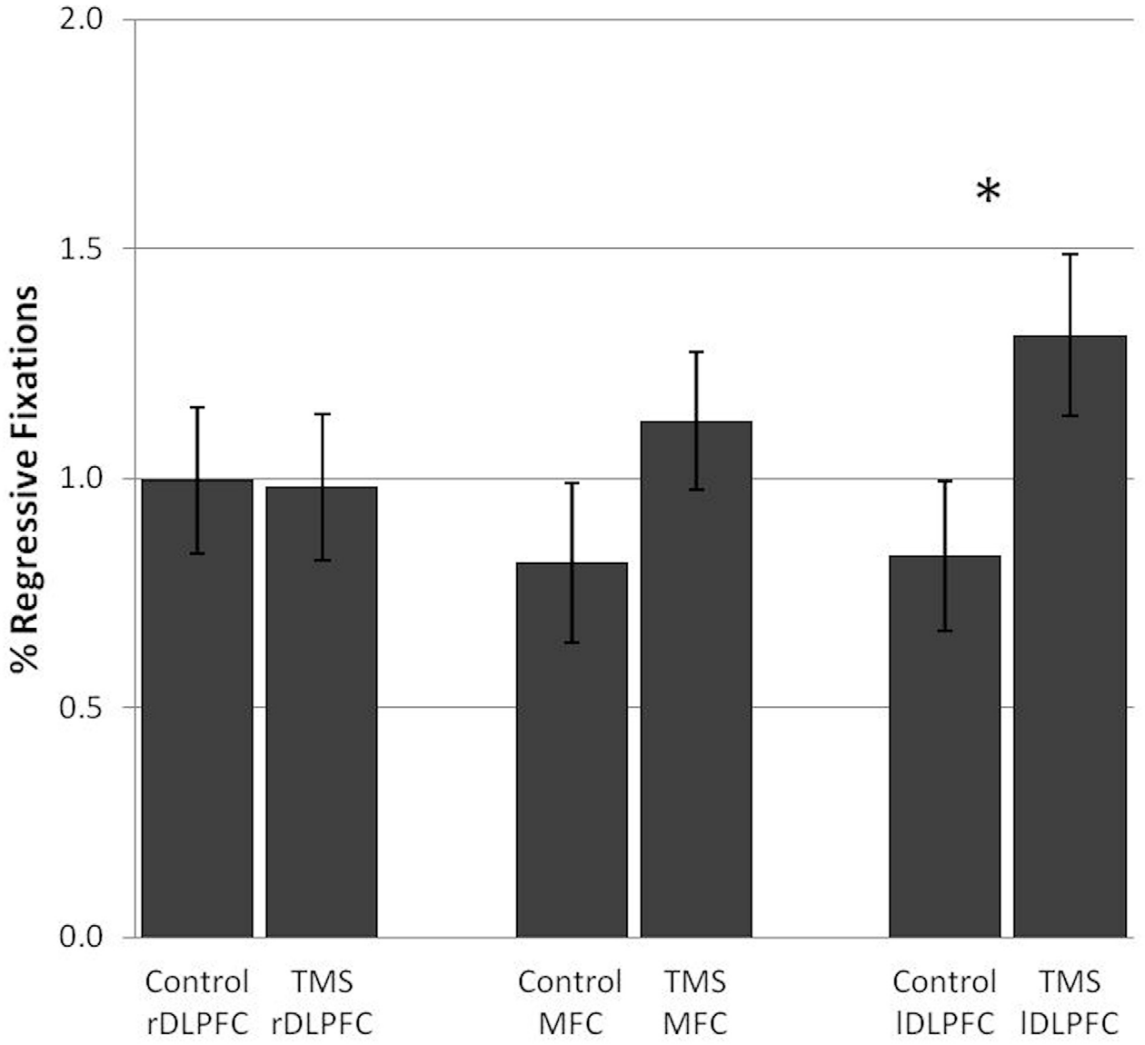
**TMS effects on the percentage of regressive fixations in the number and letter search task**. Significant differences in regressive fixations between the control and TMS condition were found after stimulation over the left dorsolateral prefrontal cortex. Error bars indicate standard errors of the mean (SE). ^*^0.021.

TMS over the lDLPFC led to a significant increase in the relative number of regressive fixations [*F*_(1, 17)_ = 6.47, *p* = 0.021] in 67% of the subjects. Also, in the lDLPFC TMS group, regressive fixations were not a bad visual strategy, but were negatively correlated with processing time (*r* = −0.012, n.s.). No change in the percentage of regressive fixations was found after stimulation over the rDLPFC [*F*_(1, 17)_ = 0.01] or the MFC [*F*_(1, 17)_ = 2.23] (Figure 5).

In sum, inhibition of both the lDLPFC and the MFC led to an increase in anticipatory fixations, considered to be a good visual strategy in this task. In the number and letter search task a TMS-induced increase in looking ahead reflects an increase in looking at a different stimulus category. This did not affect performance.

Inhibition of the left DLPFC led to an increase in regressive fixations. This increase in a *per se* bad visual strategy was observed in the face of unchanged performance.

## Discussion

This study investigated and compared the functional roles of the rDLPFC, lDLPFC and the MFC in executive functions using a TBS approach.

We observed executive functions “at work” by measuring eye movements that showed how two visual search tasks were solved. In our quest to observe efficient, strategic and goal directed visual exploration behavior, we were particularly interested in TMS-induced changes in visual strategies associated with good and poor performance in two tasks with low and high cognitive demands. While performance was unchanged, the specific pattern of modulated eye movements when confronted with a specific cognitive demand shed light on what processes were impaired as a result of TMS over the three frontal areas.

In the number search task, functional inhibition of the rDLPFC resulted in a decrease of anticipatory fixations, which was found to be a good visual strategy in control conditions. This was paired with a rDLPFC TMS-induced decrease of stimulus fixations. These effects suggest that in conditions with low executive demands, functional inhibition of the rDLPFC interfered with the anticipation of future events as well as the attention to task relevant information.

In the more demanding number and letter search task, inhibition of the lDLPFC and the MFC led to an increase in anticipatory fixations. In this task, anticipatory fixations were found to be a good visual strategy and also reflect attention to a different stimulus category. In view of unchanged performance, these effects might represent a compensatory attempt to account for TMS-induced deficits in attentional switching from one aspect of information to another. In the number and letter search task, inhibition of the lDLPFC also led to an increase in regressive fixations, a *per se* bad visual strategy. In view of unchanged performance, this might reflect a compensatory mechanism to account for lDLPFC TMS induced deficits in working memory by updating information on task progress.

While all three frontal areas are known to be involved in executive functions, their specific functional specialization is still unclear. In part, this is due to most localization studies being either strictly descriptive or correlative. Here, we offer an experimental TMS approach that can prospectively induce temporally circumscribed functional decrements in healthy subjects. TMS-induced functional decrements are homogenous among subjects and rather focal neuroanatomically, despite possible remote effects (Knoch et al., 2006; Stefan et al., 2008). Hence, it has the potential to clarify the involvement of these structures in executive functioning.

### Results in the low demand number search task

Our first task was an easy and highly overlearned task. It required searching for and performing a mouse-click on numbers in sequential order. In the absence of attentional switching or working memory demands, this low demand task was considered a suitable tool to observe basic executive functions such as mental anticipation of future events and focusing on task relevant information. Looking at stimuli that will become relevant at a later point in time was described as a major contribution (Land, 2006, 2009), even a prerequisite (Hayhoe et al., 2003) of vision to the organization and control of actions in everyday life. Also, in naturalistic tasks, irrelevant objects are hardly ever fixated, emphasizing top down, rather than bottom—up mechanisms of gaze control (Land, 2006, 2009).

In this task, TMS over the rDLPFC resulted in a decrease in anticipatory fixations, a visual strategy associated with fast performance. We interpret this as a deficit in anticipating future events, i.e., planning. These effects were paired with a rDLPFC TMS-induced deficit in paying attention to task relevant stimuli and suppress irrelevant information, an executive function also known as response selection.

In line with these results, a number of studies suggest that planning and response selection are linked. “Look-ahead fixations” were described as both a task dependent, goal-directed strategy (Pelz and Canosa, 2001) and to reflect planning (Mennie et al., 2007). In the “Tower of London” task, efficient planners directed their gaze selectively toward the problem critical balls in the workspace, whereas those who made errors spent more time looking at irrelevant items and were strongly influenced by the preceding problem (Hodgson et al., 2000). In our study, reduced planning after a functional inhibition of the rDLPFC was paralleled by more attention being paid to irrelevant information, and less attention to relevant stimuli. These findings suggest a common neural basis of planning and response selection located in the rDLPFC.

It remains unclear why the TMS-induced decrease in a visual strategy associated with good performance in control conditions did not affect performance. We speculate that the rDLPFC TMS-induced deficit might not have been strong enough to affect performance. Given the low task demands, it seems plausible that subjects were able to maintain the same performance despite the decrease in anticipatory fixations.

A large body of lesion and imaging studies provides evidence that the prefrontal cortex plays a crucial role in planning (Koechlin et al., 2000; Fincham et al., 2002; Cazalis et al., 2003; Unterrainer and Owen, 2006; Gouveia et al., 2007; Tanji et al., 2007). Basso et al. (2006) found impaired planning after rTMS application over bilateral prefrontal areas in humans. Given that neuroimaging studies have yielded bilateral prefrontal activation, a recent study addressed the functionally specific contributions of the left and right DLPFC to planning (Kaller et al., 2011). The authors found that rDLPFC activations were associated with the mental generation and evaluation of action sequences, which is perfectly in line with our own results.

A selective involvement of the dorsolateral prefrontal cortex in inhibition and response selection was found in a clinical study (Gehring and Knight, 2002), as well as fMRI and TMS studies (Rowe et al., 2000; Hadland et al., 2001). With respect to lateralization, a recent TMS study demonstrated that stimulation of the rDLPFC affected the inhibitory control of impulses in decision making (Cho et al., 2010).

To conclude, we suggest that the rDLPFC plays an important role in planning, a task dependent, top-down process aimed at acquiring information for future use by looking ahead. Planning and response selection appear to be closely related, as the specific contribution of the rDLPFC seems to lie in the mental generation of action sequences, the suppression of irrelevant information and the attention to relevant information.

### Results in the high demanding number and letter search task

In contrast to the first task, the number and letter search task is more demanding. It requires switching attention from a number sequence to a sequence of letters. The task has been described as a valid measure of the ability to alternate between cognitive categories (Olivera-Souza et al., 2000). The constant switching between a number and letter series also makes it more difficult to keep track of task progress, i.e., the task makes high working memory demands.

Stimulation over both the MFC and the lDLPFC resulted in a significant increase in anticipatory fixations in the number and letter search task. In this task, looking ahead means looking at a letter before clicking on the preceding number number and vice versa. Hence, anticipatory fixations in this task seem to be aimed at guiding attention toward a different stimulus dimension, which will become relevant next. This might facilitate attentional switching. It has been documented that during switch implementation, the relevant stimulus dimension is fixated more (Chevalier et al., 2010). In the face of unchanged performance, this suggests that the increase in anticipatory fixations—a good strategy in this task—is likely to reflect a compensatory mechanism to account for attentional switching deficits induced by TMS over the MFC and the lDLPFC.

At first glance, it seems that the increase in anticipatory fixations after inhibition of the lDLPFC and the MFC is contrary to the decrease in anticipatory fixations after inhibition of the rDLPFC (see above). This would raise the question whether these effects are due to possible remote effects of TMS on other parts of the frontal cortex. However, this interpretation does not take into account the different cognitive demands of the two tasks. Importantly, no opposing effects of TMS over the three stimulation sites were found on any visual strategy in the context of the same cognitive demands.

A crucial involvement of the MFC and the lDLPFC in attentional switching was found previously using variants of the Trail Making Test and other paradigms. A combined fMRI and TMS study showed that medial frontal regions, especially the pre—supplementary motor area is essential for task switching (Rushworth et al., 2002). These findings were confirmed and extended by investigating brain activity related to the set-shifting component of the Trail Making Test B. A marked asymmetry in favor of the left dorsolateral and MFC was found irrespective of the response modality ((Moll et al., 2002); Moll et al., using a verbal Trail Making Test; (Zakzanis et al., 2005), using a fMRI compatible writing device). Ko et al. (2008) applied continuous TBS stimulation to the left and right DLPFC in an attempt to transiently disrupt its function and establish a causal relation between observed brain activity and task performance in a PET study. Again, a significant hemispheric asymmetry was observed: inhibition of the left, but not right DLPFC impaired performance in a set-shifting task.

After TBS over the lDLPFC, subjects displayed more regressive fixations in the number and letter search task. As mentioned above, this task makes high working memory demands. The lDLPFC TMS-induced increase in this *per se* bad visual strategy left performance unchanged. This might suggest that regressive fixations were not a bad visual strategy. Indeed, in the lDLPFC TMS group, looking back was negatively correlated with processing time (even if only marginally), making it a good visual strategy rather than a bad visual strategy. We interpret these results in terms of lDLPFC stimulation induced working memory disruption, leading to more regressive fixations in an attempt to compensate for this deficit when faced with high working memory demands. When unable to hold information active in a working memory buffer, the most obvious compensatory strategy is to access this information again by looking back. Similarly, Kemper et al. (2004) reported more regressive fixations in low-span readers when processing ambiguous sentences with high working memory demands. Because of the verbal nature of our number and letter search task, we believe that the lDLPFC TMS effect is most likely to represent a compensatory strategy to account for a verbal working memory deficit in our left hemisphere language dominant subjects.

D'Esposito and Postle (1999) attempted to clarify the dependence of mnemonic processes on the prefrontal cortex. They found that lesions to the DLPFC were most likely to disrupt rehearsal processes supporting working memory. Our data is perfectly in line with these clinical findings and support the view that the lDLPFC supports verbal working memory by holding retrospective information active in a memory buffer.

After inhibition of the lDLPFC, we found both an increase in anticipatory fixations as well as an increase in regressive fixations in the number and letter search task. As described, these are likely to reflect compensatory strategies to account for TMS-induced deficits in attentional switching and working memory, respectively. These compensatory strategies are very different: Anticipatory fixations imply a memory buffer to store information about future events. They enhance the working memory load, instead of lowering it. In contrast, attempts to deal with a high working memory load are more likely to result in a decrease in anticipatory fixations, paired with an increase in regressive fixations. This pattern was found in violin players in an attempt to reduce the information load in the memory buffer (Wurtz et al., 2009). Our results thus suggest that the MFC and the lDLPFC form a functional unit that (independent of other cognitive functions) supports attentional switching. Another functional unit limited to the lDLPFC seems to support working memory.

To conclude, we suggest that the right DLPFC specifically contributes to executive functioning by two related mechanisms: First, it suppresses attention to irrelevant information and guides attention toward task relevant information. Second, it plays a crucial role in anticipating future events. In contrast, the left DLPFC together with the MFC seem to support the switching of attention from one aspect to another by guiding attention toward the relevant information during switch implementation. The specific contribution of the left DLPFC in executive functioning lies in keeping information active in a working memory buffer by re-accessing or rehearsing it.

Because of the explorative approach of this study, our data have to be interpreted carefully. To extend knowledge on executive functions and their functional specification, further studies should specifically address our hypotheses. The quest for the functional specification of frontal structures must include experimental approaches to expand previous knowledge from descriptive lesion studies and correlational imaging methods. Executive functions are likely to be mediated by dynamic and flexible networks instead of discrete foci (Elliott, 2003). A concept of executive functions as adaptive and flexible behavior calls for more direct behavioral measures. We have demonstrated that eye movements (unlike classical time measures) have the capacity to observe the human frontal cortices “at work.” In our experiments, they proved suitable to register both a decrease of efficient, task dependent strategies, as well as an adaptive, compensatory allocation of cognitive resources when faced with malfunctioning and high demands of the same executive process. Eye movements are thus a sensitive and specific measure of executive functioning.

We believe that the use of qualitative behavior data might be beneficial both with respect to advancing theoretical concepts of executive functions as well as understanding their neural basis.

### Conflict of interest statement

The authors declare that the research was conducted in the absence of any commercial or financial relationships that could be construed as a potential conflict of interest.
